# Experimental data on the behavior of a heat-generating fluid in a rotating horizontal cylinder under transverse vibrations

**DOI:** 10.1016/j.dib.2019.103834

**Published:** 2019-03-21

**Authors:** Viktor G. Kozlov, Alexei A. Vjatkin, Rustam R. Sabirov

**Affiliations:** a Perm State Humanitarian Pedagogical University, Perm, Russia; b Perm National Research Polytechnic University, Perm, Russia

## Abstract

The paper presents experimental data on the effect of transverse vibrations on a heat-generating fluid in a rotating horizontal cylinder. To obtain the data, an original experimental device was designed and manufactured. The data of temperature measurements in the cavity depending on the angular velocity of rotation of the cavity, frequency and amplitude of vibrations are presented. To estimate the intensity and structure of convection, the velocity fields of fluid motion relative to the cavity in cross sections are shown. The paper is related to a research article “Convection of a heat-generating fluid in a rotating cylindrical cavity subject to transverse vibrations” Vjatkin et al, 2019.

**Table:** Specifications table

Subject area	*Fluid mechanics*
More specific subject area	*Thermal convection of fluid in oscillating force fields*
Type of data	*Images and figures*
How data was acquired	*Temperature measurements and PIV-method for measuring the velocity of fluid flows*
Data format	*Analyzed*
Experimental factors	*Cavity rotational velocity; amplitude and frequency of translational vibrations;*
Experimental features	*The measured values are the integral temperature of the fluid on the axis of the cylinder and on the cylindrical wall, depending on the parameters of vibration and rotation; fluid velocity relative to the cavity*
Data source location	*Perm, Russia*
Data accessibility	*Data is with this article -*
Related research article	*A.A. Vjatkin, V.G. Kozlov, R.R. Sabirov, Convection of a heat-generating fluid in a rotating cylindrical cavity subject to transverse vibrations, International Journal of Thermal Sciences 137 (2019) 560–570.*https://doi.org/10.1016/j.ijthermalsci.2018.12.008*(in press)*

**Table:** 

**Value of the data** • The article describes an effective tool to control over a heat transfer in a rotating cavity, containing a non-uniformly heated fluid, by using transverse vibrations. • Experimental data may be useful in planning and analyzing technological processes occurring in microgravity conditions, for example, in orbital flight conditions, when the vibrational mechanism of the convective motion emergence is one of the main ones. • The data of the experiment is of considerable interest to the chemical industry. For example, to control chemical reactions occurring with the release of a large amount of heat. • Experimental data can be used to verify mathematical models describing the convective motion of liquids in oscillating force fields.

## 1 Data

**Fig. 1:**
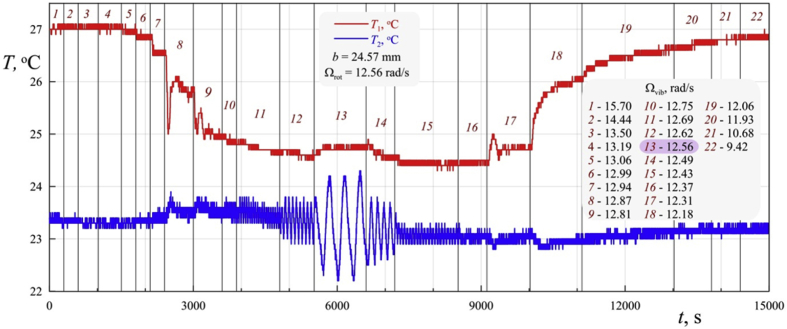
Real-time data recording from the integral temperature sensors.

Consider the data of an experiment with a heat-generating fluid in a rotating horizontal cylinder under the action of transverse vibrations [1]. At rapid rotation in the absence of vibrations, an axially symmetric temperature profile with a maximum on the axis is formed in the cylinder under the action of centrifugal inertial force. In the experiment, the angular velocity of cavity rotation ${\text{Ω}}_{\text{rot}}$ is fixed while the cyclic frequency of vibrations ${\text{Ω}}_{\text{vib}}$ changes stepwise. Fig. 1 shows a thermogram obtained from temperature sensors. Temperature $T_{1}$ is measured on the axis of the cavity, and $T_{2}$ – on the cylindrical wall. Vibrations perpendicular to the axis of rotation are able to break the equilibrium state of liquid in the case when the frequency of rotation and vibrations are close. The maximum heat transfer is achieved when the difference between the rotational and vibrational frequencies is 2–3%. (Fig. 1, steps 9 and 10 at ${\text{Ω}}_{\text{vib}}>{\text{Ω}}_{\text{rot}}$; steps 16 and 17 at ${\text{Ω}}_{\text{vib}}<{\text{Ω}}_{\text{rot}}$). At these steps, the temperature in the center of the cavity decreases, which indicates the development of flows that remove heat from the axis. At equal ${\text{Ω}}_{\text{rot}}$ and ${\text{Ω}}_{\text{vib}}$ (step 13) the low-frequency (compared with the frequency of vibration) temperature $T_{2}$ fluctuations are observed.

**Fig. 2:**
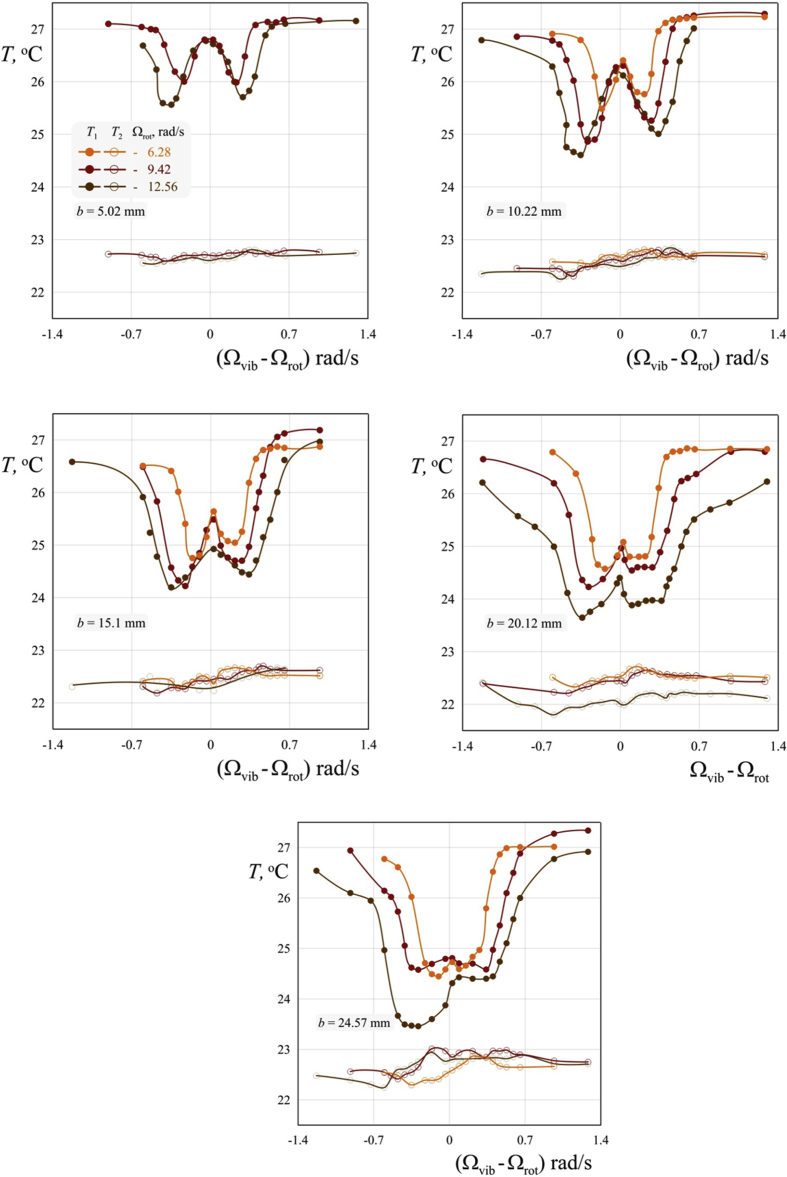
The temperature in the center of the cavity $T_{1}$ (solid symbols) and at the wall $T_{2}$ (opened symbols) versus the frequency difference ${\text{Ω}}_{\text{vib}}-{\text{Ω}}_{\text{rot}}$.

At each step of the experiment, a steady state convection mode is expected and the average temperatures $T_{1}$ and $T_{2}$ are determined. The data of experiment is shown at Fig. 2 in a form of the dependence of temperature $T_{1}$ and $T_{2}$ on the difference between frequencies ${\text{Ω}}_{\text{vib}}-{\text{Ω}}_{\text{rot}}$ and the amplitude of vibrations *b*. At each amplitude of vibrations there are 3 series of experiments with fixed values of ${\text{Ω}}_{\text{rot}}=6.28,\;9.42\;\text{and}\;12.56\;\text{rad/s.}$ Heat transfer increases with increasing the intensity of vibrations.

**Fig. 3:**
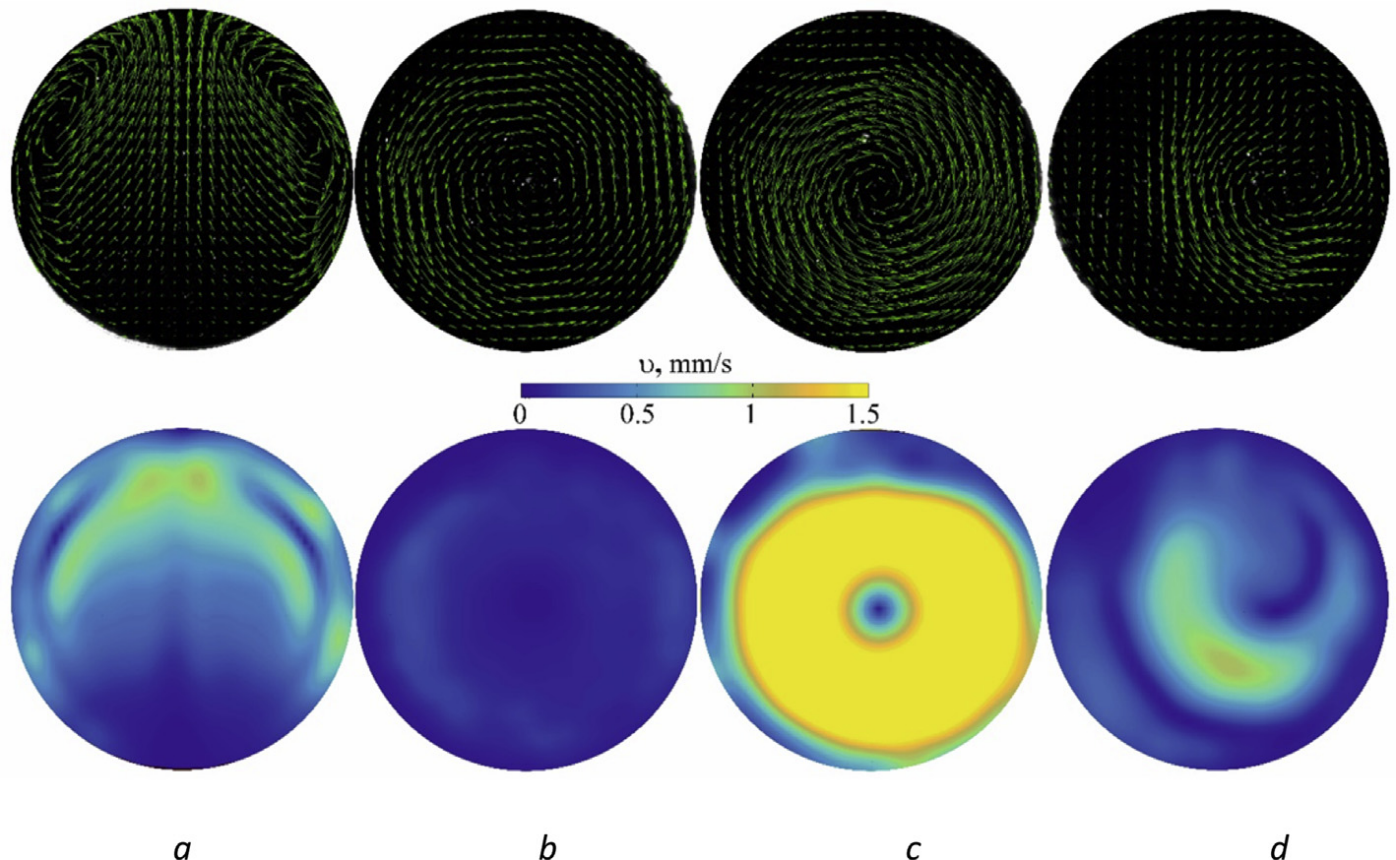
Fluid flow pattern and absolute velocity field in the cavity reference system.

The movement of fluid in the working cavity is investigated by PIV-method [2] using a high-speed camera. The data of speed measurements are shown in Fig. 3. Four cases are considered: fixed cylindrical cavity (*a*), rotating cavity (*b*), rotation and vibrations in the area of maximum heat transfer (*c*) and at ${\text{Ω}}_{\text{vib}}={\text{Ω}}_{\text{rot}}$ (*d*). The specific power of heat generation in all experiments is the same and equals 0.026 W/sm^3^. The amplitude of vibrations *b* = 10.8 mm. The maps of convective flows are built in the cavity reference system. In the case of a fixed cavity (*a*), the convective structures are a torch, that carries heat from the central part of the cavity to the upper part, and two rolls that extend along the entire cavity. The rapid rotation of the cavity with an angular velocity ${\text{Ω}}_{\text{rot}}=6.28\;\text{rad/s}$ leads to a stationary temperature distribution with a maximum on the cylinder axis. The fluid is practically in a state of mechanical quasi-equilibrium. The velocity of the fluid relative to the cavity is small (*b*).

In the case of vibrational action, the occurrence of convective flows is observed in a rotating cavity. The intensity and the structure of these flows depend on the difference between frequencies ${\text{Ω}}_{\text{vib}}-{\text{Ω}}_{\text{rot}}$. With rotation and vibrations parameters ${\text{Ω}}_{\text{rot}}=6.28\;\text{rad/s}$ and ${\text{Ω}}_{\text{vib}}=6.62\;\text{rad/s}$ maximum heat transfer is observed in the cavity; the liquid performs an intensive prograde motion relative to the cavity (Fig. 3*c*).

At equal frequencies of vibrations and rotation ${\text{Ω}}_{\text{rot}}={\text{Ω}}_{\text{vib}}$ (Fig. 3*d*) the convective flows, arising in the cavity, have a structure somewhere similar to the flow in the absence of rotation (Fig. 3*a*). One can see two 2D vortexes parallel to the cavity axis, which are stationary in the cavity frame.

## 2 Experimental design, materials, and methods

**Fig. 4:**
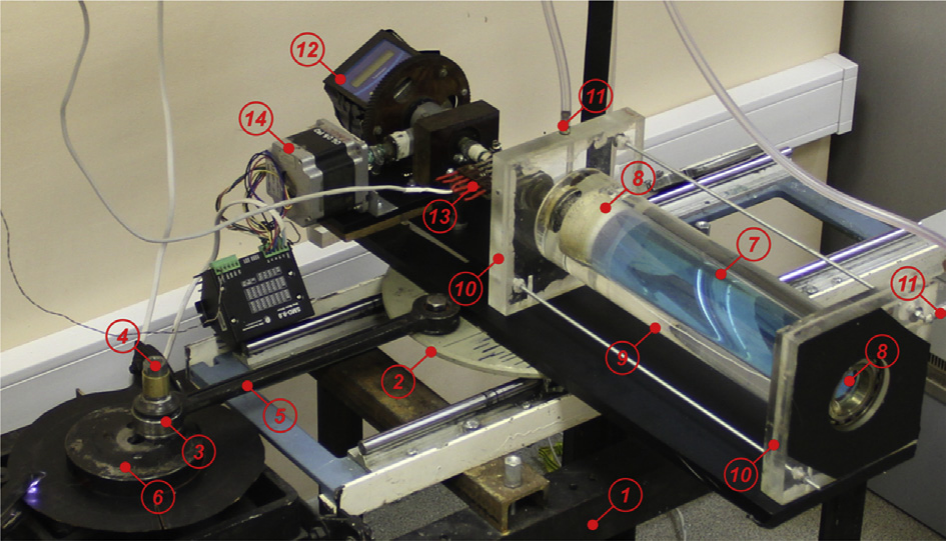
Photo of experimental complex.

Experiments are performed using a mechanical vibration stand (Fig. 4), which set the translational vibration of the rotating cavity in the horizontal direction. The massive frame *1* of the stand is fixed on the foundation, which excludes parasitic oscillations. The reciprocating motion of table *2* of the vibration stand is set by a crank mechanism *3*, which is driven by a servo drive EMG-15ASA2. The use of a servomotor ensures high accuracy of maintenance of cyclic frequency of translational oscillations ${\text{Ω}}_{\text{vib}}$. The amplitude of vibration b is determined by the position of the axis *4* of the connecting rod *5* on the rotating disk *6* and is controlled by an optical cathetometer В-630. The frequency and amplitude of vibration vary in the range $f_{\text{vib}}\equiv {\text{Ω}}_{\text{vib}}/2\pi =2-4\;\text{Hz},\;b\;=\;1-30\;\text{mm.}$ The relative error in measuring the amplitude and frequency of vibrations does not exceed 1 and 0.1%, respectively.

The working cavity *7* (Fig. 4) is a Plexiglas cylinder, closed from the ends by flanges *8*. A cylinder with an internal diameter *R* = 22 mm and length *L* = 230 mm is used in the experiments. The cavity is installed in an external fixed casing, which is made of Plexiglas and consists of a pipe *9* with an internal diameter of 100 mm and end walls 10. The working cavity is installed in the end walls *10* with the help of ball bearings and seals. This ensures its free rotation around the horizontal axis in a fixed casing. The cooling of the cylindrical border of the working cavity is carried out due to the circulation of constant temperature water coming from a powerful jet thermostat through fittings *11*.

Heat generation in the cell is provided by passing an alternating electric current. To do this, copper electrodes are attached to the inner sides of the flanges *8*, and copper sulfate is added to the liquid (not more than 5%). An alternating current source GW Instek APS-9501 is used to precisely maintain the harmonic waveform and voltage level.

Temperature measurement is carried out on the axis of the cavity ($T_{1}$) and on the cylindrical wall ($T_{2}$). The sensors are made of copper wire with a diameter of 0.02 mm, laid in the form of several loops along the entire length of the cavity along the generatrix and glued with a transparent self-adhesive film 0.1 mm thick and approximately 4 mm wide. The resistance of each sensor is 100 Ω. To isolate a thermal sensor located on the axis of the cavity, a glass capillary *7* (Fig. 4) with an outer diameter of 2.5 mm and an inner 1 mm is used. Thus, the readings of the integral sensors $T_{1}$ and $T_{2}$ characterize the average along the length of the cavity temperature on the axis of the cylinder and on its inner boundary. Distilled water is used as a working fluid. The coolant temperature is set by a thermostat and equals 19.0 °C. Temperature measurements are carried out with a multichannel device “Termodat” *12*, which rotates with the cavity and transmits data to a PC through an electrical collector *13*. Temperature measurement error does not exceed 0.1  С.

The rotation of the working cavity with an angular velocity ${\text{Ω}}_{\text{rot}}$ is set by the stepper motor FL86STH156 (*14*), which is controlled by the driver SMD-78. The instability of the rotation speed does not exceed 0.01 rps.

Experiments with heat-generating fluid in a rotating horizontal cylinder in the absence of translational vibrations were carried out earlier and published in Refs. [3], [4], [5]. The case of a cylindrical layer with boundaries of different temperatures is considered in Refs. [6], [7]. In these works, the gravity field which rotates in a reference frame connected to the cavity plays the role of an oscillating force field by analogy with the inertial force in the case of a circular oscillations of the cavity. Theoretical research in this area was carried out in Ref. [7].

## References

[1] Vjatkin A.A., Kozlov V.G., Sabirov R.R. (2019). Convection of a heat-generating fluid in a rotating cylindrical cavity subject to transverse vibrations. Int. J. Therm. Sci..

[2] Thielicke W., Stamhuis E.J. (2014). PIVlab – towards user-friendly, affordable and accurate digital particle image velocimetry in MATLAB. J. Open Res. Softw..

[3] Kozlov V.G., Ivanova A.A., Vjatkin A.A., Sabirov R.R. (2015). Vibrational convection of heat-generating fluid in a rotating horizontal cylinder. The role of relative cavity length. Acta Astronaut..

[4] Vyatkin A.A., Ivanova A.A., Kozlov V.G., Sabirov R.R. (2014). Convection of a heat-generating fluid in a rotating horizontal cylinder. Fluid Dyn..

[5] Kozlov V., Vjatkin A., Sabirov R. (2013). Convection of liquid with internal heat release in a rotating container. Acta Astronaut..

[6] Vyatkin A.A., Ivanova A.A., Kozlov V.G. (2016). Convective heat transfer in a rotating horizontal cylindrical fluid layer. J. Appl. Mech. Tech. Phys..

[7] Kozlov V.G. (2004). Thermal vibrational convection in rotating cavities. Fluid Dyn..

