# Supplementary material for: Experimental data on the behavior of a heat-generating fluid in a rotating horizontal cylinder under transverse vibrations
Source: Data Brief. 2019 Mar 21;24:103834. doi: 10.1016/j.dib.2019.103834 (PMC6454223; doi:10.1016/j.dib.2019.103834)
Supplement: Multimedia Component 1 [file mmc1.docx]

The authors, Viktor G. Kozlov, Alexei A. Vjatkin and Rustam R. Sabirov^,^ confirm the absence of conflicts of interest in the article “Experimental data on the behavior of a heat-generating fluid in a rotating horizontal cylinder under transverse vibrations” submitted for publication to journal “Data in Brief”
